# Duplication of 9p24.3 in three unrelated patients and their phenotypes, considering affected genes, and similar recurrent variants

**DOI:** 10.1002/mgg3.1592

**Published:** 2021-01-17

**Authors:** Zuzana Capkova, Pavlina Capkova, Josef Srovnal, Katerina Adamova, Martin Prochazka, Marian Hajduch

**Affiliations:** ^1^ Department of Medical Genetics University Hospital Olomouc Olomouc Czech Republic; ^2^ Department of Medical Genetics Faculty of Medicine and Dentistry Palacký University Olomouc Olomouc Czech Republic; ^3^ Institute of Molecular and Translational Medicine Faculty of Medicine and Dentistry Palacký University Olomouc Olomouc Czech Republic

**Keywords:** 9p24.3 duplication, 15q11.2 duplication, 16p11.2 duplication, developmental delay

## Abstract

**Background:**

Recent studies suggest that duplication of the 9p24.3 chromosomal locus, which includes the *DOCK8* and *KANK1* genes, is associated with autism spectrum disorders (ASD), intellectual disability/developmental delay (ID/DD), learning problems, language disorders, hyperactivity, and epilepsy. Correlation between this duplication and the carrier phenotype needs further discussion.

**Methods:**

In this study, three unrelated patients with ID/DD and ASD underwent SNP aCGH and MLPA testing. Similarities in the phenotypes of patients with 9p24.3, 15q11.2, and 16p11.2 duplications were also observed.

**Results:**

All patients with ID/DD and ASD carried the 9p24.3 duplication and showed intragenic duplication of *DOCK8*. Additionally, two patients had ADHD, one was hearing impaired and obese, and one had macrocephaly. Inheritance of the 9p24.3 duplication was confirmed in one patient and his sibling. In one patient *KANK1* was duplicated along with *DOCK8*. Carriers of 9p24.3, 15q11.2, and 16p11.2 duplications showed several phenotypic similarities, with ID/DD more strongly associated with duplication of 9p24.3 than of 15q11.2 and 16p11.2.

**Conclusion:**

We concluded that 9p24.3 is a likely cause of ASD and ID/DD, especially in cases of *DOCK8* intragenic duplication. *DOCK8* is a likely causative gene, and KANK1 aberrations a modulator, of the clinical phenotype observed. Other modulators were not excluded.

## Graphical abstract

We describe three unrelated patients with autism spectrum disorders and intellectual disabilities / developmental delay who are carriers of the 9p24.3 duplication including DOCK8 and KANK1. Clinical features of duplication are similar as 15q11.2 and 16p11.2 changes. We compared clinical features of patients with dupication 9p24.3, 15q11.2 and 16p11.2.

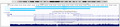

## 1 INTRODUCTION

The overlapped dedicator of cytokinesis 8 (*DOCK8*, * 611432) gene and the KN motif and ankyrin repeat domains 1 (*KANK1*, * 607704) gene are both found on chromosome 9 at locus 9p24.3. The *DOCK8* gene encodes a member of the DOCK protein family that participates in the intracellular signaling network. The product of the *DOCK8* gene is important for proper immune cell migration, synapse formation, and signal transduction (Gadea & Blangy, 2014; Su, 2010), and is also expressed in the brain, mainly in the caudate nucleus, which is engaged in proper cognitive functioning (Lin et al., 2017; Su, 2010). The *KANK1* gene encodes a member of the Kank protein family which contains multiple ankyrin repeat domains. The product of the *KANK1* gene functions in cytoskeleton formation by regulating actin polymerization (Gee et al., 2015).

Genetic duplications of 9p24.3, and therefore the *DOCK8* and *KANK1* genes, are associated with autism spectrum disorders (ASD), intellectual disability/developmental delay (ID/DD), attention deficit hyperactivity disorder (ADHD), epilepsy, learning problems, and language disorders (Engelhardt et al., 2009; Glessner et al., 2017; Griggs et al., 2008; Krgovic et al., 2018; Oikonomakis et al., 2016; Ruiter et al., 2007; Su, 2010). Loss of function of the *DOCK8* and *KANK1* genes, through deletion or sequence changes, causes an autosomal‐recessive form of the hyper‐immunoglobulin‐E (or hyper‐IgE) syndrome, cerebral palsy spastic quadriplegia type 2, and central nervous system (CNS) development disorders (Gee et al., 2015; Glessner et al., 2017; Lin et al., 2017).’

Population studies have shown that copy number variants (CNVs) of *DOCK8* or *KANK1* are not frequently detected in the general population (0.11%), although duplications of these genes are more common than deletions in the records held by the Database of Genomic Variants (DGV), the International Standards for Cytogenomic Arrays (ISCA), and the Database of Genomic Variation and Phenotype in Humans Using Ensemble Resources (DECIPHER).

Here we report findings of duplications of chromosome location 9p24.3 (i.e., the *DOCK8*/*KANK1* genes) in three unrelated patients, which provide evidence of the pathogenic impact of this duplication in patients with ID/DD, ASD, ADHD, and macrocephaly. We also compare our results with the previously published phenotypes of patients carrying 9p24.3, 15q11.2, and 16p.11.2 duplications.

## 2 MATERIALS AND METHODS

### 2.1 Editorial policies and ethical considerations

According to the Code of Ethics of the World Medical Association, informed consent was obtained from the patients’ parents prior to clinical data collection from the specialist and testing and processing of biological materials (Declaration of Helsinki, Supplemental Information S1, S2).

### 2.2 Materials

Patients with ID/DD and ASD were referred to genetics consultation at the Department of Medical Genetics, University Hospital Olomouc by specialists from the Department of Psychiatry and Pediatrics, Olomouc University Hospital. Additionally, tests and biological material from patients meeting the clinical criteria for the testing of congenital metabolic defects (e.g., eating habits) were processed in the Laboratory for Inherited Metabolic Disorders, University Hospital and Palacký University in Olomouc.

### 2.3 Methods

Genetic testing of ID/DD and ASD patients was conducted according to international recommendations (Ho et al., 2016; Richards et al., 2015). Large chromosomal aberrations (≥ 5 Mb) and aberrant repeats in the promotor of the fragile X mental retardation protein translational regulator 1 (*FMR1*), resulting from the testing, were excluded by conventional karyotyping and fragment analysis performed according to Zhou, 2006.

CNVs were tested using single nucleotide polymorphism (SNP) array for comparative genomic hybridization (aCGH; Zhou, 2006). A Cytoscan HD instrument (Affymetrix, Santa Clara, CA, USA) was used for SNP aCGH analysis according to the manufacturer's protocols. The CHAS v1.2.2 program (Affymetrix, Santa Clara, CA, USA) was used to call CNVs. Data from this analysis were stored in the Gene Expression Omnibus (GEO, https://www.ncbi.nlm.nih.gov/geo/) under number GSE114870 and GSE132453. Identified CNVs were clinically classified using curated genetics databases (ISCA, DECIPHER, SFARI, DGV) according to established guidelines (Kearney et al., 2011; Schaefer & Mendelsohn, 2013). Additionally, multiplex ligation‐dependent probe amplification (MLPA) with Probemix P385‐A2 was used to more accurately determine the extent of CNV revelation by SNP aCGH in the *DOCK8* gene. MLPA reactions were set up according to the manufacturer's protocols (MRC‐Holland, Amsterdam, Netherlands).

All PCR products were determined by capillary electrophoresis with an ABI 3130 genetic analyzer and the Gene Mapper software (Applied Biosystems, Foster City, CA) and the Coffalyser program (MRC‐Holland, Amsterdam, Netherlands) was used to call CNVs (MRC‐Holland, Amsterdam, Netherlands). Tests for connexin 26 and the Prader‐Willi syndrome (PWS, 15q11‐13) were conducted when patients met the appropriate clinical criteria (hearing impairment, obesity, etc.). Connexin 26 was analyzed using the procedure developed by Tang and colleagues, including Sanger sequencing (Tang et al., 2006). Methyl specific MLPA (MS MLPA) was used for Prader‐Willi syndrome testing using ME028‐C1, according to the manufacturer's protocol (MRC‐Holland, Amsterdam, Netherlands), and the Coffalyser program was used to call CNVs.

## 3 RESULTS

Three unrelated patients with ID/DD and ASD carried a duplication of chromosome locus 9p24.3, which includes the *DOCK8* and *KANK1* genes. CNV analysis proved the inherited origin of the 9p24.3 duplication in one patient and his sibling. This observation corresponds to the 1.75% frequency of this duplication in patients with ID/DD and ASD, and no 9p24.3 duplication was detected in patients without ID/DD in the Department of Medical Genetics, University Hospital Olomouc. Normal karyotypes and numbers of CGG triplets in the *FMR1* promoter were observed in all three patients. Aberration of Connexin 26 was excluded in one patient with hearing impairment and no genomic and methylation changes were shown in the 15q11‐13 region for two patients. Table 1 summarizes the clinical features of these patients.

**TABLE 1:** Summary of clinical and genetic observations of patients with 9p24.3 duplication

Observed feature	Patient 1	Sibling of Patient 1	Patient 2	Patient 3
Birth weight	50th percentile	10th percentile	60th percentile	N/A
Birth length	60th percentile	50th percentile	75th percentile	N/A
Height	3rd percentile	4th percentile	50th percentile	N/A
Head circumference	88th percentile	50th percentile	>97th percentile	25th percentile
Weight	>97th percentile	>97th percentile	50th percentile	3rd percentile
BMI	28.7	25.0	15.4	N/A^†^
ID/DD	yes	yes	yes	yes
ASD	yes	no	yes	yes
Additional clinical features	ADHD, hearing impairment	ADHD, dyslalia	ADHD, macrocephaly	none

Abbreviations: ADHD, attention deficit hyperactivity disorder; ASD, autism spectrum disorder; BMI, body mass index; ID/DD, intellectual disability/developmental delay; N/A, not available.

† Notes from the genetics clinic: no obesity or overweight.

### 3.1 Patient 1

Patient 1 was male, Caucasian, and of nonconsanguineous parents; the mother and father were 34 and 31 years old, respectively. The mother had been treated for epilepsy, with no reports of other monogenic diseases or neurodevelopmental disorders (ID/DD and ASD). A clinical picture of the father is unavailable. Patient 1 was the mother's third pregnancy. The pregnancy was uneventful; delivery was spontaneous during week 36 of gestation; birth weight was 2,500 g (50th percentile), length 48 cm (60th percentile).

**FIGURE 1:**
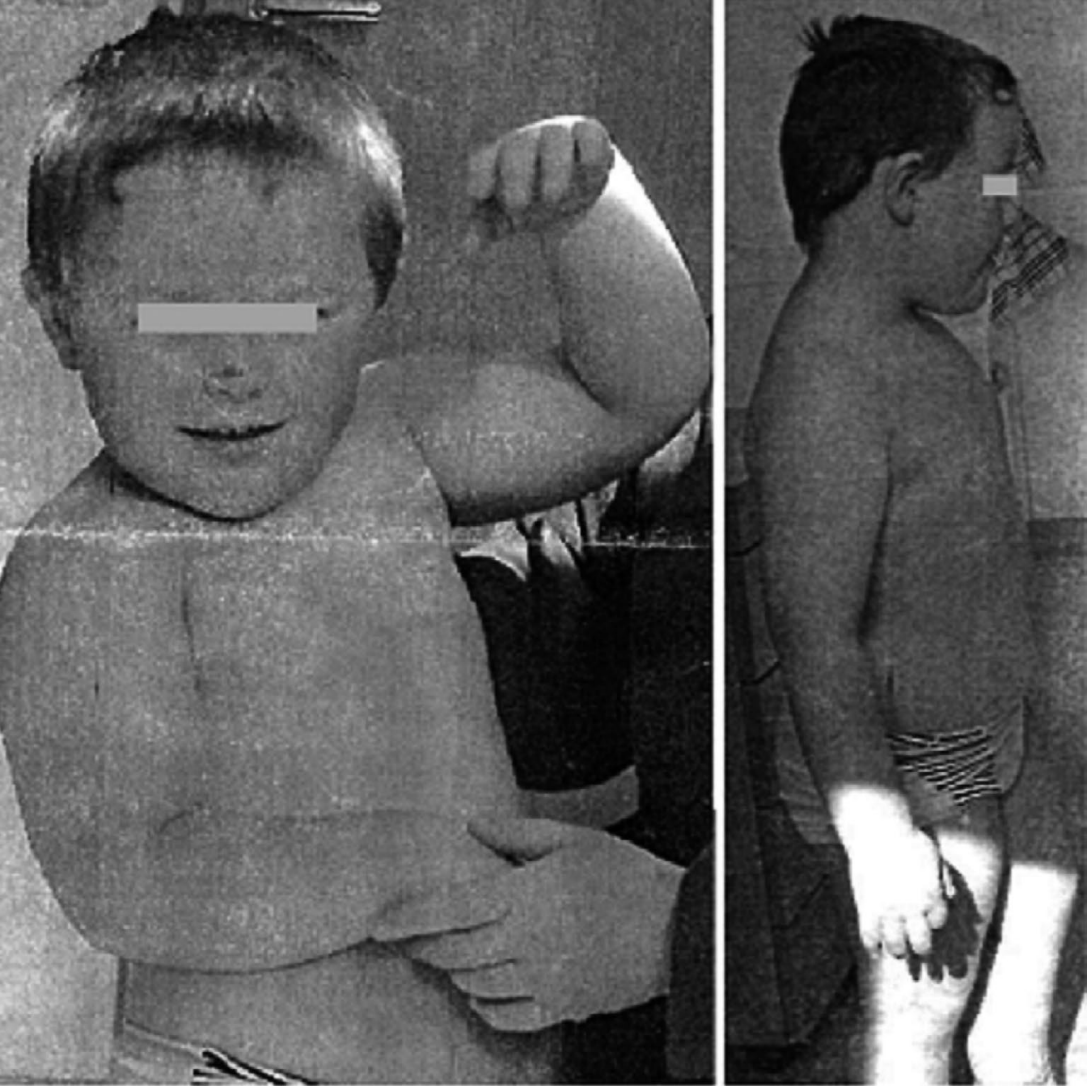
Photo of Patient 1

The boy had been treated for bronchial asthma and underwent an orchiectomy at 1 year of age. He was achieving developmental milestones normally until age 18 months when speech delay, hyperactivity, and sleep disorder were observed. Hearing impairment and ADHD were diagnosed at age 3 years. He underwent genetic testing at age 4 years for hearing impairment, ADHD, and obesity. The diagnosis of ASD with mild intellectual impairment was confirmed by a psychiatrist at age 5. Growth parameters at age 7 years were as follows: height 116 cm (3rd percentile), weight 38.6 kg (>97th percentile), BMI (body mass index) 28.7 (obese), and head circumference 54 cm (88th percentile; Figure 1). Apart from a short philtrum and broader nasal bridge, the boy exhibited no dysmorphic features (Table 1).

**FIGURE 2:**
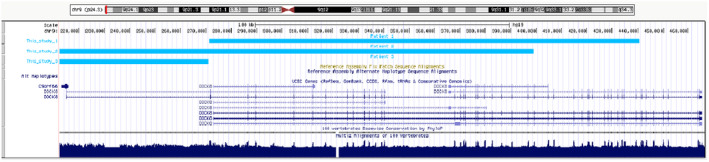
Graph showing the extent of *DOCK8* duplications in Patients 1, 2, and 3

Genetic testing of Patient 1 revealed a paternally inherited 9p24.3 duplication (Human GRCh37/hg19 chromosome 9: 271533–440683, 169 kb). MLPA with P385 probe mix determined the extent of the duplication in the *DOCK8* gene (exons 2 – 43; Table 2, Figure 2). A 35delG in the *GJB2* gene (which encodes gap junction beta‐2 protein) and the Prader‐Willi syndrome were excluded in this patient.’

**TABLE 2:** Comparison of genetics observations with literature findings

Origin of information	Identification of patient	Chr.	Coordinates (Human GRCh37/hg19)	Length (kb)	Duplicated coding genes	Duplicated exons of *DOCK8* gene	Duplicated noncoding genes	Inheritance (if known)
This work	Patient 1	9	271533–440683	169 kb	*DOCK8*	2–43^†^	none	paternal
	Patient 2	9	203861–398865 516412–664333	195, 148 kb	*DOCK8, KANK1*	1–26^†^, none	*C9orf66, RN7SL412P, RNU6‐1327P*	N/A
	Patient 3	9	1–271132	271 kb	*DOCK8, CBWD1, FOXD4*	1–2^†^	*C9orf66, DDX11L5, FAM138C, MIR1302‐9*	N/A
Krgovic et al. (2018)	Patient 1	9	204193–271316	67 kb	*DOCK8*	1	*C9orf66*	N/A
	Patient 3	9	204221–271287	67 kb	*DOCK8*	1	*C9orf66*	*de novo*
Vanzo et al. (2019)	17	9	221948–332560 408416–729135	111 kb	*DOCK8*, *KANK1*	2–9, 29–48	*RN7SL412P*, *RNU6‐1327P*	maternal
	18	9	314208–512067	198 kb	*DOCK8*, *KANK1*	7–48	*RN7SL412P*	N/A
	19	9	314208–518499	204 kb	*DOCK8*, *KANK1*	7–48	*RN7SL412P*	N/A
	20	9	314208–518675	204 kb	*DOCK8*, *KANK1*	7–48	*RN7SL412P*	N/A
	21	9	379523–742934	363 kb	*DOCK8*, *KANK1*	21–48	*RN7SL412P*, *RNU6‐1327P*	N/A
	22	9	404371–613317	209 kb	*DOCK8*, *KANK1*	27–48	*RN7SL412P*, *RNU6‐1327P*	N/A
	23	9	445993–622360	176 kb	*DOCK8*, *KANK1*	44–48	*RN7SL412P*, *RNU6‐1327P*	N/A
	24	9	460071–519546	59 kb	*DOCK8*, *KANK1*	47–48	*RN7SL412P*	N/A

Abbreviation: N/A ‐ not available.

† Extent of duplication in DOCK8 verified by MLPA.

Identification through the work of Krgovic et al. (2018).

### 3.2 Siblings of Patient 1

**FIGURE 3:**
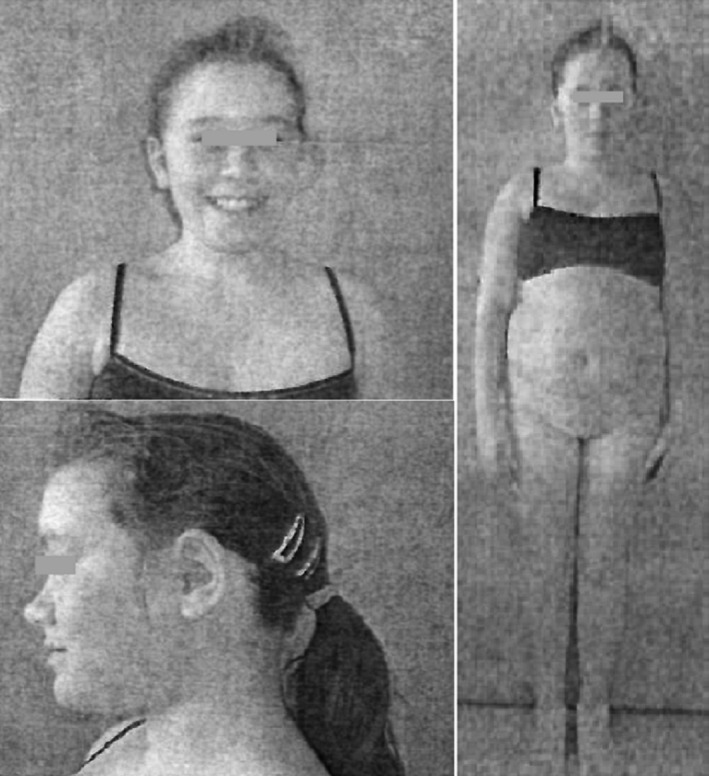
Photo of Patient 1’s sibling

The boy had two older sisters. The oldest was by a different father and was 22 years old and healthy at the time of the study. She was not genetically tested because of her good health and different father. The second sister shared the same father as Patient 1 and was 11 years old at the first visit to the Department of Medical Genetics. She was born in week 38 of pregnancy; birth weight 2300 g (10th percentile), length 50 cm (50th percentile). Apart from dyslalia, her psychomotor development was uneventful up to age 4 years when ADHD and compulsive behaviors were diagnosed by a psychiatrist. At age 11 years, she had the following parameters: height 136 cm (4th percentile), weight 45.5 kg (>97th percentile), BMI 25 (overweight), and head circumference 53 cm (50th percentile; Figure 3). She was overweight and exhibited facial features slightly like those of her brother.

The second sister of Patient 1 inherited the same 9p24.3 duplication (Human GRCh37/hg19 chromosome 9: 271533–440683, 169 kb) as her brother.

### 3.3 Patient 2

Patient 2 was male, Caucasian, and the first child of nonconsanguineous parents; the mother and father were 29 and 38 years old, respectively. The mother had latent tetany but was being treated with medication; she also had a family history of Bekhterev's disease and Raynaud syndrome. The father had been treated for Crohn's disease. No other monogenic diseases or neurodevelopmental disorders were reported in the families of either parent. Patient 2 had no siblings, and neither parent had ID/DD and ASD. Patient 2 was the mother's second pregnancy. The mother experienced syncope and olfactory aura during the third trimester of her pregnancy, perhaps the equivalent of an epileptic seizure causing olfactory aura and syncope but without convulsions. Delivery was in gestational week 37 by Caesarean section (mother's choice); birth weight was 2950 g (60th percentile), length 50 cm (75th percentile).

Patient 2 was achieving developmental milestones normally until age 15 months when speech delay, stereotyped movements, negativistic and aggressive behaviors, and hyperactivity were observed. The diagnosis of ASD with ADHD was confirmed by a psychiatrist at age 3 years and the child was referred to genetic counselling. Growth parameters at age 3.5 years were as follows: height 102 cm (50th percentile), weight 16 kg (50th percentile), BMI 15.4 (normal weight), head circumference 53 cm (>97th percentile). The boy was suspected of having hypoacusis (not investigated at that time). He also met the clinical criteria for testing congenital metabolic disorders which were excluded (amino acid and fatty acid metabolism and organic aciduria).

**FIGURE 4:**
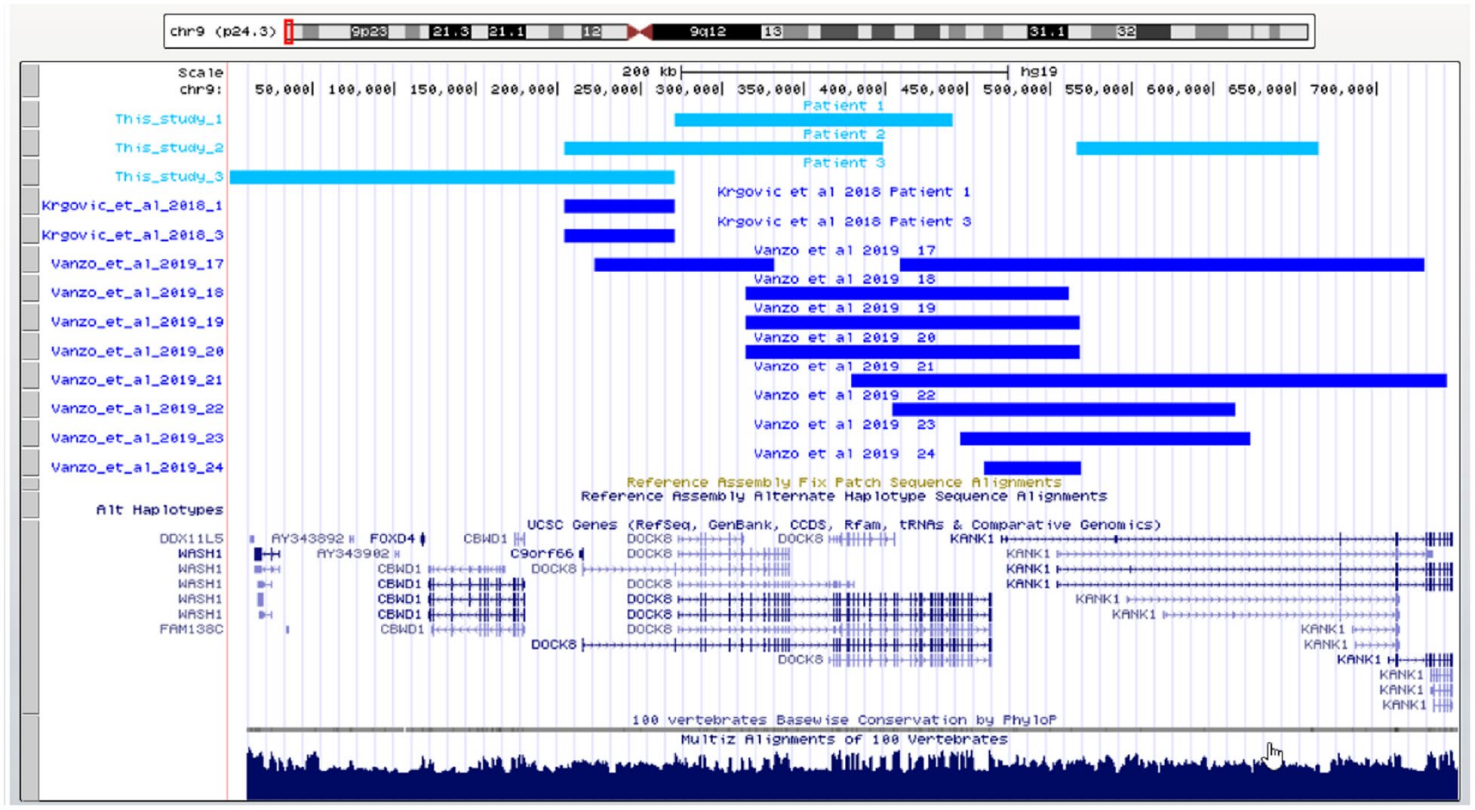
Graph showing a comparison of the description of a 9p24.3 duplication with the duplications in Patients 1, 2, and 3

Patient 2 carried two separate 9p24.3 duplications (Human GRCh37/hg19 chromosome 9: 203861–398865, 516412–664333, 195 kb, 148 kb). MLPA with P385 probe mix determined the extent of duplication in the *DOCK8* gene; exons 1 – 26 of this gene were amplified. Moreover, duplication in Patient 2 affected the coding gene *KANK1* and some noncoding genes (*C9orf66*, *RN7SL412P*, *RNU6*‐*1327P*; Table 2, Figures 2 and 4). Genetic material from the parents was unavailable and the origin of these variants is unknown. Additionally, Patient 2 demonstrated mosaic duplication (Human GRCh37/hg19 chromosome 9: 203861–1735677, 1532 kb, copy number [CN] state =2.26). This 1532‐kb mosaic duplication included protein coding genes *DOCK8*, *DMRT1* (i.e., *double sex and mab*‐*3 related transcription factor 1*), *DMRT2*, *DMRT3*, and *KANK1*, and some noncoding genes (*C9orf66*, *RN7SL412P*, *RNU6*‐*1327P*, *RNU6*‐*1073P*, *RPS27AP14*, *and RNA5SP279*). Prader‐Willi syndrome was excluded in Patient 2.

### 3.4 Patient 3

Patient 3 was male, Caucasian, and the first child of nonconsanguineous parents aged 30 (mother) and 31 (father) years. The mother was treated for cyclic bleeding disorders. No other monogenic diseases or neurodevelopmental disorders (ID/DD and ASD) were reported for the parents. Patient 3 was an only child and was fetus B of a multiple pregnancy. Fetus A underwent fetal death spontaneously. The delivery was without complications. His birth weight and length were unavailable.

**FIGURE 5:**
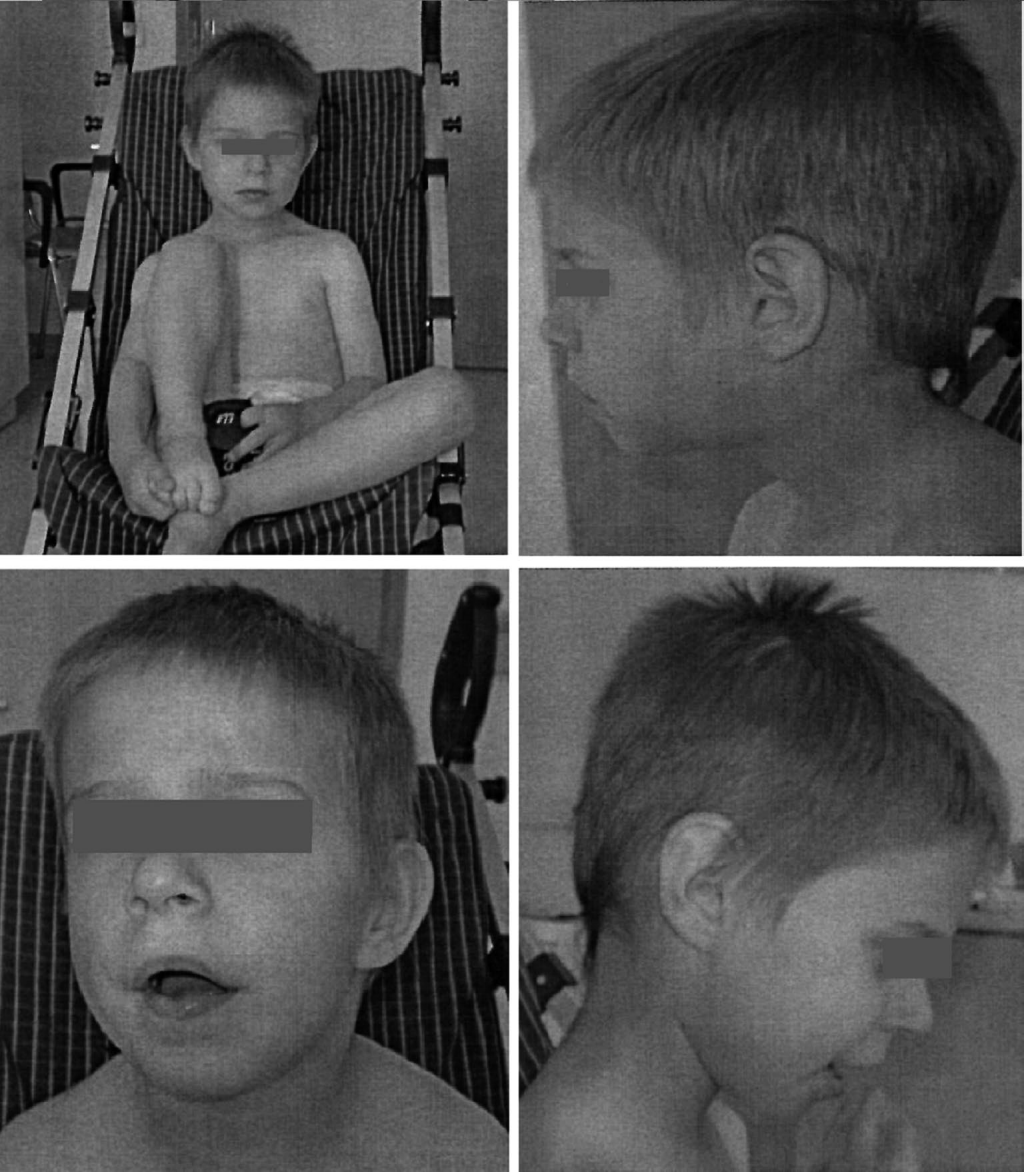
Photo of Patient 3

Newborn adaptation was normal. At age 13 months, Patient 3 underwent neurologist counselling for delays in all developmental areas. Significant developmental delays, intellectual disability, mild paleocerebellar aphasia, and incontinence were observed at age 4.5 years. ASD was diagnosed by a specialist in the psychiatric department based on autistic features such as the absence of speech and lack of understanding of communication as well as avoidance of social contact. At this age, the boy weighed 14 kg (3rd percentile) and had a head circumference of 50 cm (25th percentile). No facial dysmorphic features were observed (Figure 5). He was also referred to urology for testicular retention and to orthopedics for flat feet. Negative results were observed in magnetic resonance imaging of his brain as well as congenital metabolic disorders screening.

Patient 3 carried 9p24.3 duplication (Human GRCh37/hg19 chromosome 9: 1–271132, 271 kb), overlapped coding genes *DOCK8*, *CBWD1*, *FOXD4*, and noncoding genes *C9orf66*, *DDX11L5*, *FAM138C*, *and MIR1302*‐*9*. The origin of the 9p24.3 duplication in Patient 3 is still unknown because genetic material from the parents was unavailable (Table 2, Figures 2, 4).

## 4 DISCUSSION

Recent reports indicate that CNVs of 9p24.3, including *DOCK8* and *KANK1*, have clinical importance (Engelhardt et al., 2009; Glessner et al., 2017; Griggs et al., 2008; Krgovic et al., 2018; Oikonomakis et al., 2016; Ruiter et al., 2007; Su, 2010). However, carriers of these CNVs presented with different phenotypes, especially between deletions and duplications (Glessner et al., 2017; Jing et al., 2014; Krgovic et al., 2018; Qin et al., 2016; Singh et al., 2017). Duplications of 9p24.3 are two times more frequent than deletions in the general population (0.07% vs 0.03%) and in subpopulations of patients with ASD and ID/DD (0.46% vs 0.23%), but these ratios are not significant (*p* = 0.09, *p* > 0.05; Vanzo et al., 2019; Krgovic et al., 2018). Deletion of 9p24.3 is accompanied by more serious phenotypes than duplication; microcephaly and ADHD, specifically, appeared more frequently in carriers of deletions than of duplications (*p* = 0.01 and *p* = 0.01, respectively; Vanzo et al., 2019). Additionally, 9p24.3 duplications are inherited more often than deletions as demonstrated by observations of significantly more frequently inherited duplications than *de novo* mutations (*p* = 0.04, *p* < 0.05; Table 3; DECIPHER). In our study, we observed one patient and his sister who inherited 9p24.3 duplication from their father, but the father was unavailable for psychiatric examination and no evidence about his health exists. Inheritance information was also unavailable for the remaining patients we observed.

**TABLE 3:** Comparison of clinical phenotypes in 15q11.2, 16p11.2, and 9p24.3 duplications and deletions

CNV		CNV in population	CNV in ASD and ID/DD patients	Inherited CNV	*De Novo* CNV	ASD in CNV	ID/DD in CNV	Macrocephaly in CNV	Microcephaly in CNV	ADHD in CNV
Duplication	15q11.2	104/160 000 (0.07%) (Ulfarsson, 2017)	62/31516 (0.20%) (Moreno, 2013)	3/14 (21.43%) (Urraca et al., 2013)	11/14 (78.57%) (Urraca et al., 2013)	18/44 (40.91%) (Burnside, 2011)	19/49 (18.37%) (Burnside, 2011)	3/107 (2.80%) (Wegiel, 2012)	18/107 (16.82%) (Wegiel, 2012)	18/44 (40.91%) (Burnside, 2011)
	16p11.2	4/13696 (0.03%) (Moreno, 2013)	70/32587 (0.21%) (Girirajan, 2012)	75/180 (41.67%) (D'Angelo et al., 2016)	31/180 (17.22%) (D'Angelo, 2016)	26/114 (22.81%) (Niarchou, 2019)	36/114 (31.58%) (Niarchou, 2019)	2/76 (2.63%) (Steinman, 2016)	13/76 (17.11%) (Steinman, 2016)	48/114 (42.11%) (Niarchou, 2019)
	9p24.3	16/22054 (0.07%) (Vanzo, 2019)^†^	2/439 (0.46%) (Krgovic, 2018)	12/14 (85.71%) (DECIPHER)^‡^	2/14 (14.29%) (DECIPHER)^‡^	6/16 (37.50%) (Vanzo, 2019)^†^	13/16 (81.25%) (Vanzo, 2019)^†^	0/16 (0.00%) (Vanzo, 2019)^†^	1/16 (6.24%) (Vanzo, 2019)^†^	2/16 (12.50%) (Vanzo, 2019)^†^
Deletion	15q11.2	71/160 000 (0.04%) (Ulfarsson, 2017)	103/25113 (0.41%) (De Wolf, 2013)	32/72 (44.44%) (Cafferkey et al., 2014)	3/30 (10.00%) (Cafferkey et al., 2014)	43/171 (25.15%) (Cox, 2015)	126/172 (73.26%) (Cox, 2015)	5/71 (7.04%) (Cox, 2015)	14/71 (19.72%) (Davis et al., 2019)	28/80 (35.00%) (Cox, 2015)
	16p11.2	7/13696 (0.05%) (Moreno, 2013)	115/32587 (0.35%) (Girirajan, 2012)	67/317 (21.14%) (D'Angelo et al., 2016)	144/317 (45.43%) (D'Angelo et al., 2016)	41/217 (18.89%) (Niarchou, 2019)	61/217 (28.11%) (Niarchou, 2019)	14/83 (16.87%) (Steinman, 2016)	4/83 (4.82%) (Steinman, 2016)	63/217 (29.03%) (Niarchou, 2019)
	9p24.3	7/22054 (0.03%) (Vanzo, 2019)^†^	1/439 (0.23%) (Krgovic, 2018)	5/7 (71.43%) (DECIPHER)^‡^	2/7 (28.57%) (DECIPHER)^‡^	3/7 (42.86%) (Vanzo, 2019)^†^	4/7 (57.14%) (Vanzo, 2019)^†^	0/7 (0.00%) (Vanzo, 2019)^†^	1/7 (14.29%) (Vanzo, 2019)^†^	1/7 (14.29%) (Vanzo, 2019)^†^

Abbreviations: ADHD, attention deficit hyperactivity disorder; ASD, autism spectrum disorders; CNV, copy number variant; ID/DD, intellectual disability/developmental delay.

† Included patients with deletion/duplication of DOCK8 without two or more hits.

‡ Included duplication deletion/duplication of DOCK8 without two or more hits and in the range of GRCh37/hg19 chr9:1–1411809.

We observed carriers of 9p24.3 duplications with phenotype and penetrance similar to those seen in carriers of 15q11.2 and 16p11.2 duplications. The population frequencies of these three duplications ranging from 0.03% to 0.07% are statistically the same (*p* > 0.05; Table 3). Notably, the population frequencies of these three duplications ranging from 0.03% to 0.05% are also the same (*p* > 0.05; Table 3; Vanzo et al., 2019; Ulfarsson et al., 2017; Moreno‐De‐Luca et al., 2013). Interestingly, 9p24.3 deletion was excluded from increases in the percentages of deletions and duplications in patient subpopulations with ASD and ID/DD (frequencies ranging from 0.20% to 0.46%; *p* = 0.15; Vanzo et al., 2019; Krgovic et al., 2018). The role of 9p24.3 deletions in the ASD and ID/DD phenotype is still under discussion, but this phenotype appears strongly dependent on deletions of the *DOCK8* and/or *KANK1* genes. Additionally, recent work by Wallis and colleagues confirmed that the benign character is associated with gene deletions that overlap only *KANK1* (Tassano et al., 2016; Wallis et al., 2020).’

**FIGURE 6:**
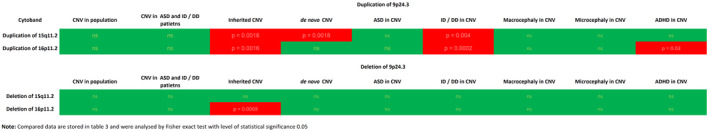
Graphical display of significant differences in phenotype between 9p24.3, 15q11.2, and 16p11.2 duplications and 9p24.3, 15q11.2, and 16p11.2 deletions. The data for comparison are presented in Table 3 and were analyzed using the Fisher exact test with a p value of 0.05 representing statistical significance

Duplications of 9p24.3 accompany ASD as often as duplications of the 15q11.2 and 16p11.2 loci (Girirajan et al., 2012; Krgovic et al., 2018; Moreno‐De‐Luca et al., 2013). However, ID/DD appear more frequently in 9p24.3 duplications than in 15q11.2 and 16p11.2 duplications (*p* < 0.05 and *p* < 0.05, respectively; Table 3, Figure 6; Niarchou et al., 2019; Cox & Butler, 2015; Vanzo et al., 2019; Burnside et al., 2011). A study by Vanzo and colleagues showed that 37.50% of 9p24 duplication carriers expressed ASD and 81.25% expressed ID/DD (Vanzo et al., 2019). These findings correspond with our observations (Table 3), as all the patients we observed were affected by ID/DD and ASD but the sister of patient 1 did not present an ASD phenotype. Also, patient 1, his sibling, and patient 2 presented with ADHD but patient 3 did not. Carriers of the 9.p24.3 and 15q11.2 duplications present with ADHD at the same frequency, ranging from 12.50% to 40.91%, but less frequently than carriers of the 16p11.2 duplication (*p* = 0.03; Table 3, Figure 6; Niarchou et al., 2019; Vanzo et al., 2019; Cox & Butler, 2015; Burnside et al., 2011).

Carriers of 9.p24.3 duplications presented with microcephaly and macrocephaly at the same frequency as that with which patients with 15q11.2 and 16p11.2 duplications present (Figure 6; Steinman et al., 2016; Vanzo et al., 2019; Wegiel et al., 2012). In this study, only patient 2 had macrocephaly and his duplication overlapped the *DOCK8* and *KANK1* genes, unlike the remaining patients who carried an intragenic duplication of *DOCK8* only. Additionally, mosaic duplication involving the *DOCK8*, *DMRT1*, *DMRT2*, *DMRT3*, *and KANK1* genes were observed in patient 2, which may explain the presence of macrocephaly in this patient. We interpret this result carefully, however, because the SNP aCGH method does not accurately distinguish between the germinal and mosaicist forms of duplications appearing in the same region.

We further analyzed the genetic material from our patients using a more detailed method (MLPA) since SNP aCGH cannot detect intragenic changes. MLPA revealed partial duplication of *DOCK8* in all patients observed. We hypothesized that partially overlapping duplications of *DOCK8* might interrupt a reading frame. Only five cases of intragenic duplication of *DOCK8* were described in the DECIPHER v9.21 database, and these patients’ phenotypes included ID/DD (3/5, 60.00%), ASD (1/5, 20.00%), and no macrocephaly, microcephaly or ADHD (DECIPHER v9.21).

The *DOCK8* gene is evolutionarily conserved (https://genome.ucsc.edu, Figure 5) and its protein product plays a role in the immune response. *DOCK8* SNPs are associated with brain tissue by expression quantitative trail loci analysis (Glessner et al., 2017, https://www.proteinatlas.org), yet ribonucleic acid and protein expression of *DOCK8* is relatively low in the brain (https://www.ncbi.nlm.nih.gov, https://www.proteinatlas.org). Moreover, *DOCK8* is topologically associated with *KANK1*, *BWD1*, *DMRT3*, and *FOXD4*, and its aberration could lead to their deregulation (Glessner et al., 2017). Although recent studies confirmed the benign character of *KANK1* deletions (Tassano et al., 2016; Wallis et al., 2020), we believe that *KANK1* duplications may play a role in phenotype modulation because *DOCK8* and *KANK1* are often duplicated together and this tandem duplication usually has more serious phenotype (Vanzo et al., 2019; Krgovic et al., 2018; DECIPHER). *KANK1* is involved in controlling cytoskeleton formation by regulating actin polymerization (Gee et al., 2015, https://www.proteinatlas.org).

Indeed, other modulatory pathways were not excluded and some of these may be similar to 15q11.2 and 16p11.2 duplications, for example, dosage‐sensitive pathways, the presence of a protective allele, sequence changes, and the impact of duplicated noncoding genes (Benítez‐Burraco et al., 2017; Jing et al., 2014; Lee et al., 2015; Ulfarsson et al., 2017; Zwaag et al., 2009). We emphasize the importance of future analyses, particularly messenger ribonucleic acid/protein expression studies and more detailed methods for revealing the start and end of duplications of 9p24.3 and other loci.

## 5 CONCLUSION

This study provides evidence of the pathogenic role of the 9p24.3 duplication. Additionally, we observed similar clinical phenotypes in carriers of the 9p24.3, 15q11.2 and 16p11.2 duplications in population frequency, frequency in ASD and ID/DD patients, microcephaly, and macrocephaly. We also observed a stronger association of the ID/DD phenotype with the 9p24.3 duplication than with the 15q11.2 and 16p11.2 duplications. Moreover, we accede to the idea that *DOCK8* is a likely causative gene of the clinical phenotypes observed and concluded that *KANK1* aberrations may modulate these phenotypes. Finally, we emphasize the need for detailed genetic testing in patients with duplications of 9p24.3 as well as of 15q11.2 and 16p11.2.

## CONFLICT OF INTEREST

The authors declare no conflicts of interest.

## AUTHOR CONTRIBUTIONS

**Zuzana Capkova**: Conceived and designed the analysis, Collected the data, Performed the analysis, Wrote the paper. **Pavlina Capkova**: Conceived and designed the analysis, Collected the data, Performed the analysis, Wrote the paper. **Josef Srovnal**: Contributed data, Performed the analysis, Wrote the paper. **Katerina Adamova**: Contributed data, Performed the analysis, Wrote the paper. **Martin Prochazka**: Contributed data, Wrote the paper. **Marian Hajduch**: Contributed data, Wrote the paper.

## Supporting information

Supplemental_ S1_Form_of_consent_CZ

Supplemental_ S2_Form_of_consent_EN

## Data Availability

The data that support the findings of this study are openly available in Gene Expression Omnibus at https://www.ncbi.nlm.nih.gov/geo/, reference number GSE114870 and GSE132453.
