# Supplementary material for: Duplication of 9p24.3 in three unrelated patients and their phenotypes, considering affected genes, and similar recurrent variants
Source: Mol Genet Genomic Med. 2021 Jan 17;9(3):e1592. doi: 10.1002/mgg3.1592 (PMC8104183; doi:10.1002/mgg3.1592)
Supplement: Supplementary file 1 — Supplemental_ S1_Form_of_consent_CZ [file MGG3-9-e1592-s002.pdf]

Informovaný souhlas pacienta (zákonného zástupce) s odběrem, uchováním a genetickým vyšetřením biologického materiálu

Pacient –<br>jméno a příjmení:	Rodné číslo<br>(číslo pojištěnce):
Datum narození:<br>(není-li rodné číslo)	Kód zdravotní<br>pojišťovny:
Adresa trvalého pobytu pacienta:<br>(případně jiná adresa)	
Jméno zákonného zástupce<br>(opatrovníka):	Rodné číslo:

Název výkonu

Odběr a uchování biologického materiálu a provedení genetického laboratorního vyšetření.

Účel genetického laboratorního vyšetření

- ☐ Ověření/potvrzení diagnózy nemoci:…………………………………………………………………………
- ☐ Zjištění predispozice pro nemoc:..……………………………………………………………………………
- ☐ Zjištění přenašečství pro nemoc:..……………………………………………………………………………
- ☐ Zjištění nemoci nebo vývojové vady u plodu:………………………………………………………………….
- ☐ Vyšetření cirkulující fetální DNA v krvi matky pro onemocnění:…………………………………………….
- ☐ Preimplantační diagnostika:……………………………………………………………..…………………
- ☐ K optimalizaci léčby:…………………………………………………………………..……………………

Povaha vyšetření

Vyšetření biologického materiálu ke zjištění geneticky podmíněných onemocnění.

Předpokládaný prospěch vyšetření

Znalost příčiny genetického onemocnění může vést k upřesnění diagnózy, možnosti léčby a předcházení možných komplikací. V případě zjištění nemoci u plodu se rodiče mohou rozhodnout o narození nebo nenarození dítěte s genetickým onemocněním. Při zjištění predispozice pro nemoc je navrženo sledování specialisty k odhalení onemocnění v časném stádium.

Alternativa vyšetření

Neexistuje.

Možné následky vyšetření

1. Pozitivní výsledek genetické analýzy může ovlivnit pacienta i další členy rodiny v riziku.
2. Zjištění tzv. neočekávaných nálezů, které mohou mít pro pacienta a osoby s ním geneticky příbuzné závažný dopad (např. zjištění přenašečství některých genetických onemocněním nebo nepotvrzení otcovství).
3. Zjištění nálezů, které se odlišují od nálezů běžných, ale jejich konkrétní dopad na současný a/nebo budoucí zdravotní stav pacienta a geneticky příbuzné osoby nelze na základě současných znalostí stanovit.

PROHLÁŠENÍ LÉKAŘE A PACIENTA

A. Prohlášení lékaře

Prohlašuji, že jsem vyšetřované/mu (zákonnému zástupci vyšetřovaného) jasně a srozumitelně vysvětlil(a) účel, povahu, předpokládaný prospěch, následky i možná rizika výše uvedeného genetického laboratorního vyšetření. Seznámil(a) jsem vyšetřovanou osobu (zákonného zástupce) i s možnými riziky a důsledky v případě odmítnutí tohoto vyšetření. Výsledky laboratorního vyšetření budou důvěrné a nebudou bez souhlasu vyšetřované osoby/zákonného zástupce sdělovány třetí straně, pokud platné právní předpisy neurčují jinak.

**Jméno a příjmení lékaře**, který podal informaci	**Podpis lékaře,** který podal informaci	**Datum**	**Hodina**

B. Prohlášení pacienta/zákonného zástupce

Potvrzuji, že mi bylo poskytnuto genetické poradenství ke genetickému laboratornímu vyšetření za účelem jak uvedeno shora. Vše mi bylo sděleno a vysvětleno jasně a srozumitelně. Měl/a jsem možnost vše si řádně, v klidu a v dostatečně poskytnutém čase zvážit, měl(a) jsem možnost se lékaře zeptat na vše, co jsem považoval(a) za pro mne podstatné a potřebné vědět a probrat s ním vše, čemu jsem nerozuměl(a). Na tyto mé dotazy jsem dostal(a) jasnou a srozumitelnou odpověď.

B.1 Za účelem výše uvedeným souhlasím s následujícím:

pozn. Vaši odpověď zakroužkujte:		
1. S odběrem vzorku biologického materiálu a s provedením genetického vyšetření.	ANO	NE
2. Se skladováním mého vzorku DNA v bance laboratoře pro další analýzy provedené k mému prospěchu, a to za předpokladu, že budu před dalším vyšetřením informován(a) a nově navrhovaná vyšetření budou provedena až s mým aktuálním informovaným souhlasem.	ANO	NE
3. S anonymním využitím DNA k lékařské vědě a výzkumu	ANO	NE
4. S využitím výsledků genetického laboratorního vyšetření a relevantních informací o mém zdravotním stavu, včetně fotodokumentace, k vědeckým a výukovým účelům za podmínky, že tyto údaje budou prezentovány a publikovány pouze v anonymní formě.	ANO	NE

B.2 Dále si přeji následující:

pozn. Vaši odpověď zakroužkujte:		
Abych s výsledky genetického laboratorního vyšetření byl(a) seznámen(a)	ANO	NE
Abych s výsledky neočekávaných nálezů byl(a) seznámen(a)	ANO	NE

Aby o výsledku vyšetření a/nebo neočekávaných nálezech byly informovány následující osoby:

SOUHLAS PACIENTA

Na základě těchto informací prohlašuji, že souhlasím s odběrem vzorku, uložením a s provedením genetického vyšetření s podmínkami uvedenými výše.

Jsem si vědom(a), že svůj souhlas mohu kdykoliv odvolat.

V Olomouci, **datum**	**Hodina**	**Podpis pacienta** (zákonného zástupce)
Vztah zákonného zástupce k pacientovi:		

Pokud se pacient/ka nemůže podepsat, uveďte důvody, pro které se pacient/ka nemohl(a) podepsat:

Jak pacient/ka projevil(a) svou vůli:

**Jméno a příjmení zdravotnického pracovníka/svědka**	**Podpis zdravotnického pracovníka/svědka**	**Datum**	**Hodina**
