# Supplementary material for: Duplication of 9p24.3 in three unrelated patients and their phenotypes, considering affected genes, and similar recurrent variants
Source: Mol Genet Genomic Med. 2021 Jan 17;9(3):e1592. doi: 10.1002/mgg3.1592 (PMC8104183; doi:10.1002/mgg3.1592)
Supplement: Supplementary file 2 — Supplemental_ S2_Form_of_consent_EN [file MGG3-9-e1592-s001.pdf]

FAKULTNÍ NEMOCNICE OLOMOUC
University hospital Olomouc
I. P. Pavlova 6, 775 20 Olomouc
Tel. 588 441 111, E-mail: fn@fnol.cz
IČO: 00098892

MEDICAL GENETICS DEPARTMENT

Agreement of Investigated Person (Legitimate Representative) with Genetic Laboratory Examination

Name of investigated person:	Personal identification number:
Date of birth: (if different from Personal ID No.)	Medical Insurance Company No.:
Patient's permanent address: (or other address)	
Name of legitimate representative:	Personal identification number:

Name of the examination: **Genetic Laboratory Examination**

The purpose of laboratory examination

- ☐ Verification/confirmation of disease diagnosis: ………………………………………………………………
- ☐ Determination of disease predisposition:…………………………………………………………………
- ☐ Determination of disease transmission: ………………………………………………………………………
- ☐ Determination of foetus's disease: …………………………………………………………………………

Nature of the examination

The examination requires a collection of a tissue sample (usually venous blood) with subsequent laboratory investigation of genetically determined diseases.

Expected benefit of this examination

Knowledge of the exact cause of genetic disease allows for determining diagnosis, administering correct treatment and preventing possible complications. When the diagnosis is established in a fetus the parents can decide the future of the pregnancy. When a predisposition to genetic disease is found, follow up by appropriate specialists allows the disease to be diagnosed at an earlier stage which can improve the prognosis.

Alternative examinations

No alternative examinations.

Possible risks of the selected procedure

Bruising can develop in the site of the venepuncture.

Consequences of the procedure

1. Nausea can develop as a consequence of venepuncture in some individuals.
2. Positive result of DNA analysis can have consequences for patient and other family members who are at risk.

Proclamation of physician and investigated person

A. Proclamation of physician

I proclaim that I explained above stated genetic laboratory examination's purpose, nature, expected benefit, consequences and possible risks clearly and understandably to the person investigated (legitimate representative). I also got acquainted the person investigated with possible result/s and effects of the fact that the examination for the above stated purpose would not be possible to perform (it would not be successful) or the examination would not have evidence capacity necessary for the observed purpose fulfilment. I got acquainted the person investigated (legitimate representative) with possible risks and consequences in case of this examination's refusal. Laboratory examination results will be confidential and they will not be conveyed to the third party without consent of the person investigated (legitimate representative), unless legal regulations in force determine otherwise.

Name of physician, who instructed the patient	**Signature of the physician,** who instructed the patient	Date:	Hour:

B. Proclamation of investigated person / legitimate representative

I confirm that I was provided with the genetic counselling concerning the laboratory examination for the purpose mentioned above. I was informed on all facts clearly and understandably. I had the opportunity to consider all aspects properly, calmly and in sufficient time granted, I had the opportunity to ask the physician about everything I considered substantial and necessary for me to know and to discuss everything I did not understand. These questions of mine were answered clearly and understandably.

B. 1 For the purpose mentioned above, I agree with taking of below mentioned sample from my body and with performing of these examinations:

Cytogenetic examination:

☐ Caryotype (chromosomes analysis)
☐ Other…………………………………………………………………………………………………

Molecular genetic examinations:

☐ Disease examination: ……………………………………………………………………………………......
☐ Other: ………

Other examinations:

☐ ....………

From the sample:

☐ venous blood ☐ umbilical cord blood ☐ amniotic fluid ☐ placenta ☐ saliva ☐ tissue: derma, muscle
☐ other: ………

B. 2 Furthermore, I wish the following:

**How to complete (mark):**	**Delete where not applicable**	YES	~~NO~~
That I would be apprised of the genetic laboratory examination results		YES	NO
That the following persons would be informed about the examination results:			
I agree with eventual recoding of my person in the registry of patients		YES	NO
with the disease::…………………………………………………………………………………………			

B. 3 I decided that after the test completion, the sample will be disposed of in this way:

☐ If this is possible, my sample(s) will be stored for further analysis performed to my benefit and benefit of my family, but I will always be instructed prior to further examination and newly proposed genetic laboratory examinations will be performed with my actual informed consent.

☐ After the genetic laboratory examination, my sample(s) will be disposed of causing the risk that, in case of necessity, the examination result can not be re-verified new material taking will be necessary for further genetic tests.

I agree with anonymous DNA utilization for the medical research	YES	NO

Other: ....……………………………………………………………………………………………

Proclamation of investigated person / legitimate representative

On the basis of this instruction I proclaim that I agree with the relevant sample taking from my body and with performing of above described genetic laboratory examination under conditions mentioned above.
I am aware that I can withdraw my agreement at any time. .

Olomouc, date	Hour	**Signature of investigated person (legitimate representative)**
Relation to investigated person:		

Drafted by: The Society for Medical Genetics JEP
Correction: MUDr. Jiří Hyjánek, Ph.D., MUDr. Václava Curtisová
Head Physician: doc. MUDr. Ishraq Dhaifalah, PhD.
Representative for Healthcare: MUDr. Jiří Hyjánek, Ph.D.
